# Interleukin-6 Expression under Gravitational Stress Due to Vibration and Hypergravity in Follicular Thyroid Cancer Cells

**DOI:** 10.1371/journal.pone.0068140

**Published:** 2013-07-02

**Authors:** Xiao Ma, Markus Wehland, Ganna Aleshcheva, Jens Hauslage, Kai Waßer, Ruth Hemmersbach, Manfred Infanger, Johann Bauer, Daniela Grimm

**Affiliations:** 1 Institute of Biomedicine, Pharmacology, Aarhus University, Aarhus, Denmark; 2 Clinic for Plastic, Aesthetic and Hand Surgery, Otto-von-Guericke-University, Magdeburg, Germany; 3 Institute of Aerospace Medicine, German Aerospace Centre (DLR), Cologne, Germany; 4 Max-Planck Institute for Biochemistry, Martinsried, Germany; Northwestern University Feinberg School of Medicine, United States of America

## Abstract

It is known that exposing cell lines *in vitro* to parabolic flights changes their gene expression and protein production patterns. Parabolic flights and spaceflight in general are accompanied by transient hypergravity and vibration, which may impact the cells and therefore, have to be considered too. To estimate the possible impact of transient hypergravity and vibration, we investigated the effects of these forces separately using dedicated ground-based facilities. We placed follicular thyroid ML-1 and CGTH W-1 cancer cells in a specific centrifuge (MuSIC Multi Sample Incubator Centrifuge; SAHC Short Arm Human Centrifuge) simulating the hypergravity phases that occur during one (P1) and 31 parabolas (P31) of parabolic flights, respectively. On the Vibraplex device, the same cell lines were treated with vibration waves corresponding to those that occur during a whole parabolic flight lasting for two hours. After the various treatments, cells were harvested and analyzed by quantitative real-time PCR, focusing on the genes involved in forming (*ACTB*, *MYO9*, *TUBB*, *VIM*, *TLN1*, and *ITGB1*) and modulating (*EZR*, *RDX*, and *MSN*) the cytoskeleton, as well as those encoding growth factors (*EGF*, *CTGF*, *IL6*, and *IL8*) or protein kinases (*PRKAA1* and *PRKCA*). The analysis revealed alterations in several genes in both cell lines; however, fewer genes were affected in ML-1 than CGTH W-1 cells. Interestingly, *IL6* was the only gene whose expression was changed in both cell lines by each treatment, while *PKCA* transcription remained unaffected in all experiments. We conclude that a PKCa-independent mechanism of *IL6* gene activation is very sensitive to physical forces in thyroid cells cultured *in vitro* as monolayers.

## Introduction

*In vivo* tumors comprise neoplastic cells, non-malignant stromal cells, and migratory hematopoietic cells [1]. Different tumors are dominated by phenotypically and functionally heterogeneous cancer cells, which guide the complex interactions between the cell types and regulate tumor growth, progression, metastasis, and angiogenesis [2]. Thus, neoplastic or cancer cells are the major constituent of malignant tumors. Their analysis may indicate possible ways of malignant tumor development and treatment.

We focused on follicular thyroid carcinomas, which are malignant epithelial tumors expressing follicular patterns. Normally, they are encapsulated [3]–[5]. *In vivo*, the neoplastic cells driving the progress of cancer are epithelial cells at different stages of dedifferentiation. Neoplastic thyroid follicular cancer cells are represented by the lines ML-1, FTC-133 and CGTH-W1 for this study [5]–[7]. In recent years, thyroid cancer cells have been shown to be affected incubated on a Random Positioning Machine (RPM) or a clinostat, devices developed to simulate microgravity on Earth [8]–[12]. We found changes in the two- to three-dimensional growth of thyroid cancer cells cultured on the RPM, accompanied by an alteration in the concentration of various proteins and the expression of a considerable number of genes [8]–[12].

The RPM is a device designed to simulate microgravity on Earth. For this purpose, samples are rotated around all three spatial directions in a random manner. In the course of the experiment, the direction of the gravity vector changes constantly and its effects might be cancelled out over time [13]. The altered behavior of the cancer cells incubated on this machine may be due to altered gravity (simulated microgravity). To prove this, we exposed thyroid cancer cells and endothelial cells to short-term real microgravity generated in aircrafts during parabolic flights. Exposure to real microgravity led to similar, but not identical, results compared to the RPM experiments [14], [15]. These differences may be due to the fact that the RPM does not simulate microgravity for our chosen cell system and investigated parameters, or that microgravity is interrupted by hypergravity phases and accompanied by vibrations. During a parabolic flight, each of the 31 parabolas normally flown includes 22 s of microgravity and periods of 1 *g* and 1.8 *g*, as well as vibration caused by the engine [14], [16].

To investigate the complex mechanical effects affecting the cells during a parabolic flight, it is important to characterize the influence of short-term hypergravity and vibration on cells without exposing them to microgravity. Thus, we performed separate hypergravity and vibration simulation tests, using methods that simulated the acceleration profile of one or 31 parabolas, as well as the vibrations occurring during the whole flight. Moreover, we focused on the expression of the cytokines IL-6 and IL-8 as well as protein kinases.

## Methods

### Cell Culture

The human thyroid cancer cell lines ML-1 [5] and CGTH-W1 [6] were seeded in T75 cm^2^ or T25 cm^2^ culture flasks and fed RPMI 1640 medium (Invitrogen, Eggenstein, Germany) supplemented with 10% fetal bovine serum (Biochrom, Berlin, Germany), 100 units penicillin/mL, and 100 µg streptomycin/mL, and grown until confluence.

### Hypergravity Experiments

Hypergravity was generated using the Multi Sample Incubator Centrifuge (MuSIC, DLR, Cologne, Germany), which was placed in an incubator at 37°C and 5% CO_2_. Driven by a special computer program, cells were exposed to a hypergravity profile that occurs during one parabola (P1) and 31 parabolas (P31). This device was used to treat the cells, whose mRNA was determined subsequently. Confluently grown cells from T75 cell culture flasks were trypsinized and transferred to 5-mL tubes. The tubes were filled with cell culture medium and the cells allowed to equilibrate before centrifugation. Corresponding to the fixation times of the cells during a parabolic flight, the cells were exposed either to one cycle of two 20-s-long 1.8 *g* phases interrupted by a 22-s pause (P1) or to 2 h lasting 1.8 *g* phases (P31). In addition, we performed experiments on the Short Arm Human Centrifuge (SAHC, DLR, Cologne, Germany), with cells from T75 cell culture flasks due to the high amount of material needed for the analysis. On this device, we exposed cells to a continuous hypergravity phase of about 2 hours corresponding to 31 parabolas. We collected n = 5 static 1 *g* controls and n = 5 1.8 *g* hyper-*g* samples for Western blot analyses (n = 5; P31), as well as n = 5 static 1 *g* controls (P1), n = 5 static 1 *g* controls (P31), and n = 5 1.8 *g* hyper-*g* (P1 and P31) for real-time PCR, respectively. The 1 *g* controls were grown in parallel in a neighboring identical incubator.

### Vibration Experiments

T25 culture flasks containing 90% confluent monolayers were fixed on the Vibraplex platform in an incubator at 37°C with 5% CO_2_ in air and treated according to a protocol published earlier [14]. Briefly, applying Vibraplex, the cells were exposed to vibrations comparable to those occurring during parabolic flights [16]. Frequencies ranging from 0.2 Hz to 14 kHz were adjusted, corresponding to the three phases: pull up (1.8 *g*), free fall (microgravity, µ*g*), and pull out (1.8 *g*), as recorded and analyzed by Schmidt [16] over approximately 2 hours, which is how long the 31 parabolas of real parabolic missions last. Afterwards, the medium was removed and the cells scraped off and collected in 3 ml of cold phosphate buffered saline (PBS). After a subsequent centrifugation (4000 rpm), the pellet was stored at −80°C for Western blot analysis and PCR.

The 1 *g* controls were grown separately in the same incubator. We collected n = 5 static 1 *g* controls and n = 5 2-hour vibration samples for Western blot analyses (n = 5; P31) as well as n = 5 samples for real-time PCR, respectively.

### RNA Isolation

Cells for quantitative real-time PCR were fixed with RNA*later* (Applied Biosystems, Darmstadt, Germany) at a ratio of 4∶1. Subsequently, the flasks were stored at 4°C. Immediately before use, the RNAlater was replaced by PBS (Invitrogen, Darmstadt, Germany). The cells were scraped off using cell scrapers (Sarstedt, Nümbrecht, Germany), transferred to tubes and pelleted by centrifugation (2500×*g*, 10 min, 4°C). The RNeasy Mini Kit (Qiagen, Hilden, Germany) was used according to the manufacturer’s instructions to isolate total RNA. RNA concentrations and quality were determined spectrophotometrically at 260 nm using a NanoDrop instrument (Thermo Scientific, Wilmington, DE, USA). The isolated RNA had an A260/280 ratio of >1.5.

cDNA for the quantitative real-time PCR was then obtained with the First Strand cDNA Synthesis Kit (Fermentas, St. Leon-Rot, Germany) using 1 µg of total RNA in a 20-µL reverse transcription reaction mixture.

### Quantitative Real-time PCR

Quantitative real-time PCR was used to determine the expression levels of the genes of interest. The Primer Express® software was utilized to design appropriate primers with a T_m_ of about 60°C (Table 1).

**Table 1:** Primers used for quantitative real-time PCR.

Gene	Primer Name	Sequence
*18S rRNA*	18S-F	GGAGCCTGCGGCTTAATTT
	18S-R	CAACTAAGAACGGCCATGCA
*ACTB*	ACTB-F	TGCCGACAGGATGCAGAAG
	ACTB-R	GCCGATCCACACGGAGTACT
*CTGF*	CTGF-F	ACAAGGGCCTCTTCTGTGACTT
	CTGF-R	GGTACACCGTACCACCGAAGAT
*EGF*	EGF-F	TGCCAGCTGCACAAATACAGA
	EGF-R	TCTTACGGAATAGTGGTGGTCATC
*EZR*	EZR-F	GAAGTGCACAAGTCTGGGTACCT
	EZR-R	CTCCCACTGGTCCCTGGTAAG
*IL6*	IL6-F	CGGGAACGAAAGAGAAGCTCTA
	IL6-R	GAGCAGCCCCAGGGAGAA
*IL8*	IL8-F	TGGCAGCCTTCCTGATTTCT
	IL8-R	GGGTGGAAAGGTTTGGAGTATG
*ITGB1*	ITGB1-F	GAAAACAGCGCATATCTGGAAATT
	ITGB1-R	CAGCCAATCAGTGATCCACAA
*MSN*	MSN-F	GAAATTTGTCATCAAGCCCATTG
	MSN-R	CCATGCACAAGGCCAAGAT
*MYO9B*	MYO9B-F	GAAATTCATCCAAGTCAGCTACCTAGA
	MYO9B-R	TGAGACACCAGGCGAGACTTT
*PRKAA1*	PRKAA1-F	AATCCTTCTGTGATTAGCCTTTTG
	PRKAA1-R	GATCCTCAGGAAAGAGATATTTTGGAA
*PRKCA*	PRKCA-F	TGGGTCACTGCTCTATGGACTTATC
	PRKCA-R	CGCCCCCTCTTCTCAGTGT
*RDX*	RDX-F	GAAAATGCCGAAACCAATCAA
	RDX-R	GTATTGGGCTGAATGGCAAATT
*TUBB*	TUBB-F	CTGGACCGCATCTCTGTGTACTAC
	TUBB-R	GACCTGAGCGAACAGAGTCCAT
*VIM*	VIM-F	TTCAGAGAGAGGAAGCCGAAAAC
	VIM-R	AGATTCCACTTTGCGTTCAAGGT

All sequences are given in 5′-3′ direction.

The primers were synthesized by TIB Molbiol (Berlin, Germany). All assays were run on a StepOnePlus Real-Time PCR System using the Power SYBR®Green PCR Master Mix (both Applied Biosystems, Darmstadt, Germany). The reaction volume was 25 µL, including 1 µL of template cDNA and a final primer concentration of 500 nM. PCR conditions were as follows: 10 min at 95°C, 40 cycles of 30 s at 95°C and 1 min at 60°C, followed by a melting curve analysis step (temperature gradient from 60°C to 95°C with +0.3°C per cycle). If all amplicons showed one single T_m_ similar to the one predicted by the Primer Express software, the PCR reactions were considered specific. Every sample was measured in triplicate and we applied the comparative C_T_ (ΔΔC_T_) method for the relative quantification of transcription levels. 18S rRNA was used as a housekeeping gene to normalize our expression data.

### Western Blot Analysis

After the treatment, samples for Western blot analysis were fixed by adding ethanol up to a final concentration of 70%. For analysis, SDS-PAGE, immunoblotting and densitometry were carried out on six replicates following routine protocols [17]–[19]. Antibodies against the following antigens were used: α-tubulin, pan-actin, β-actin, moesin and ezrin (the dilutions were 1∶1000, except for pan-actin, 1∶4000). All antibodies were purchased from Cell Signaling Technology Inc. (MA, USA). For the densitometric quantification of the bands, the stained membranes were scanned and analyzed using the Image J (http://rsb.info.nih.gov/ij/) software [20]. Since no suitable protein was found that could serve as a loading control under the investigated experimental conditions, we carefully loaded equal amounts of protein (40 µg in 10 µL) onto each gel lane and normalized the densitometric data to this value.

### STRING 9.0 Network Analysis

The proteins investigated were tabulated. For each protein, the UniProtKB entry number and gene name was acquired in UniProtKB and these names were used for the network generation with STRING 9.0 (www.string-db.org) [21]. The UniProtKB entry numbers were inserted into the input form as “multiple proteins” and “Homo sapiens” was selected as the organism. The resulting network view was downloaded as a.jpg image.

### Statistical Analysis

All statistical analyses were performed using the SPSS 16.0 software (SPSSS, Inc, Chicago, IL, USA). We employed either one-way ANOVA or the Mann-Whitney-U test where applicable. Differences were considered significant at the level of *p*<0.05. All data are represented as means ± standard deviation.

## Results

### Selected Genes and Proteins

**Figure 1:**
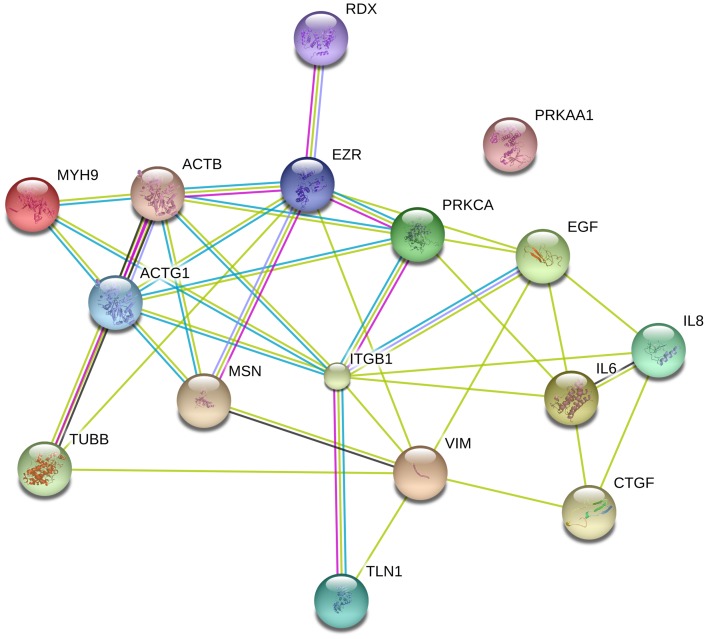
STRING analysis of the proteins tested.

To test the influence of vibration and hypergravity on cell behavior, we investigated two thyroid cancer cell lines. We assessed cytoskeletal proteins because we had previously observed that the cytoskeleton was affected during parabolic flights [14]. Thus, we focused on genes and proteins involved in forming (actin, myosin, tubulin, vimentin, talin, and integrin) and modulating (ezrin, radixin, and moesin) the cytoskeleton. Moreover, we explored those encoding growth factors (IL-6, IL-8, EGF, and CTGF) and protein kinases (PRKCA and PRKAA1). Despite the functional diversity of the proteins, they form a network of interactions (Fig. 1), with the exception of PRKAA1, the catalytic subunit of AMP-activated protein kinase (AMPK) that plays a key role in regulating cellular energy metabolism.

### Variation in mRNA Expression Induced by Vibration

**Figure 2:**
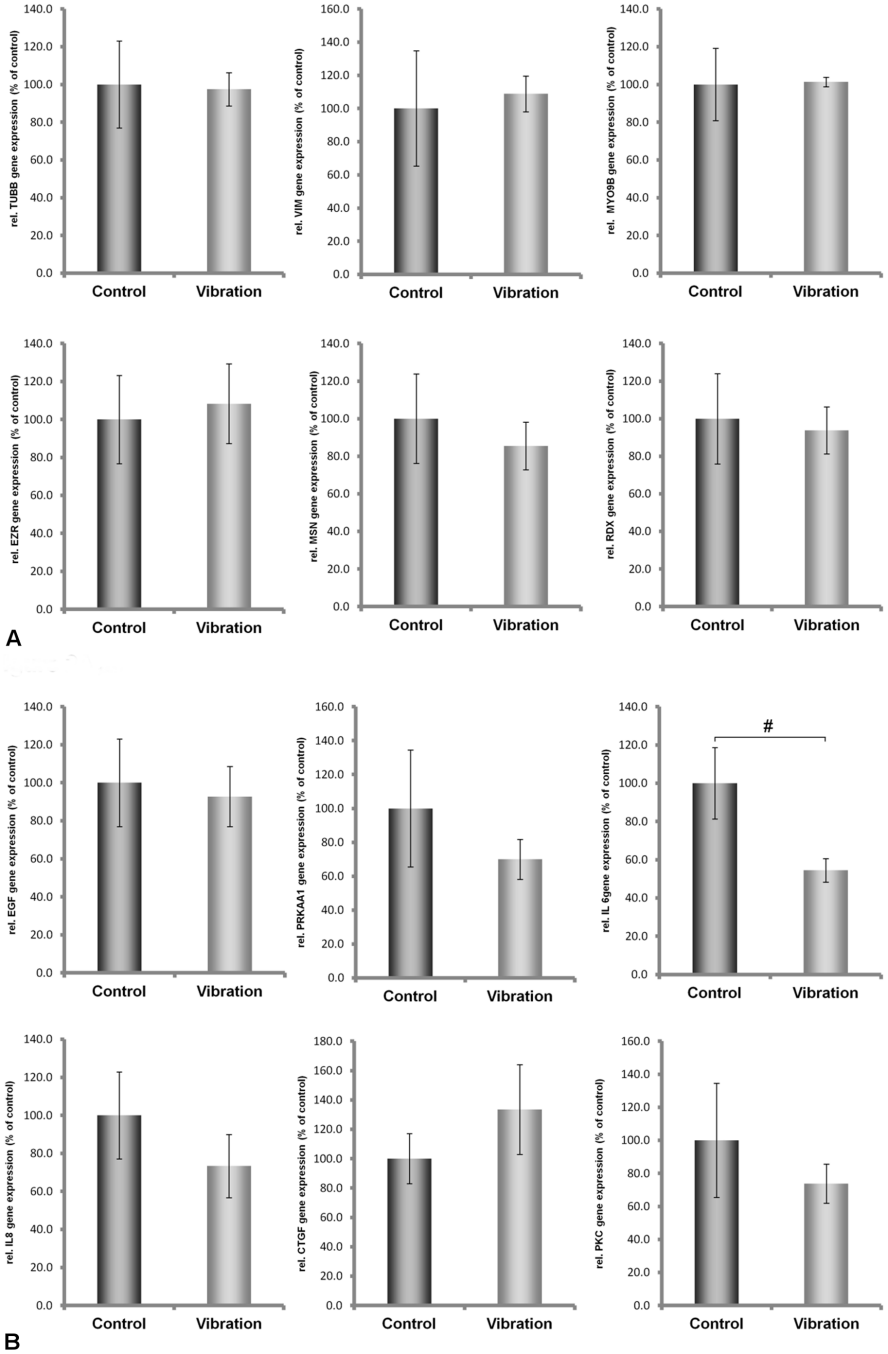
Quantitative real-time PCR showing the alterations in MYO9, TUBB, VIM, EZR, RDX, MSN, IL-6, IL-8, EGF, CTGF, PRKAA1 and PRKCA gene expression after Vibraplex treatment of ML-1 cells.

**Figure 3:**
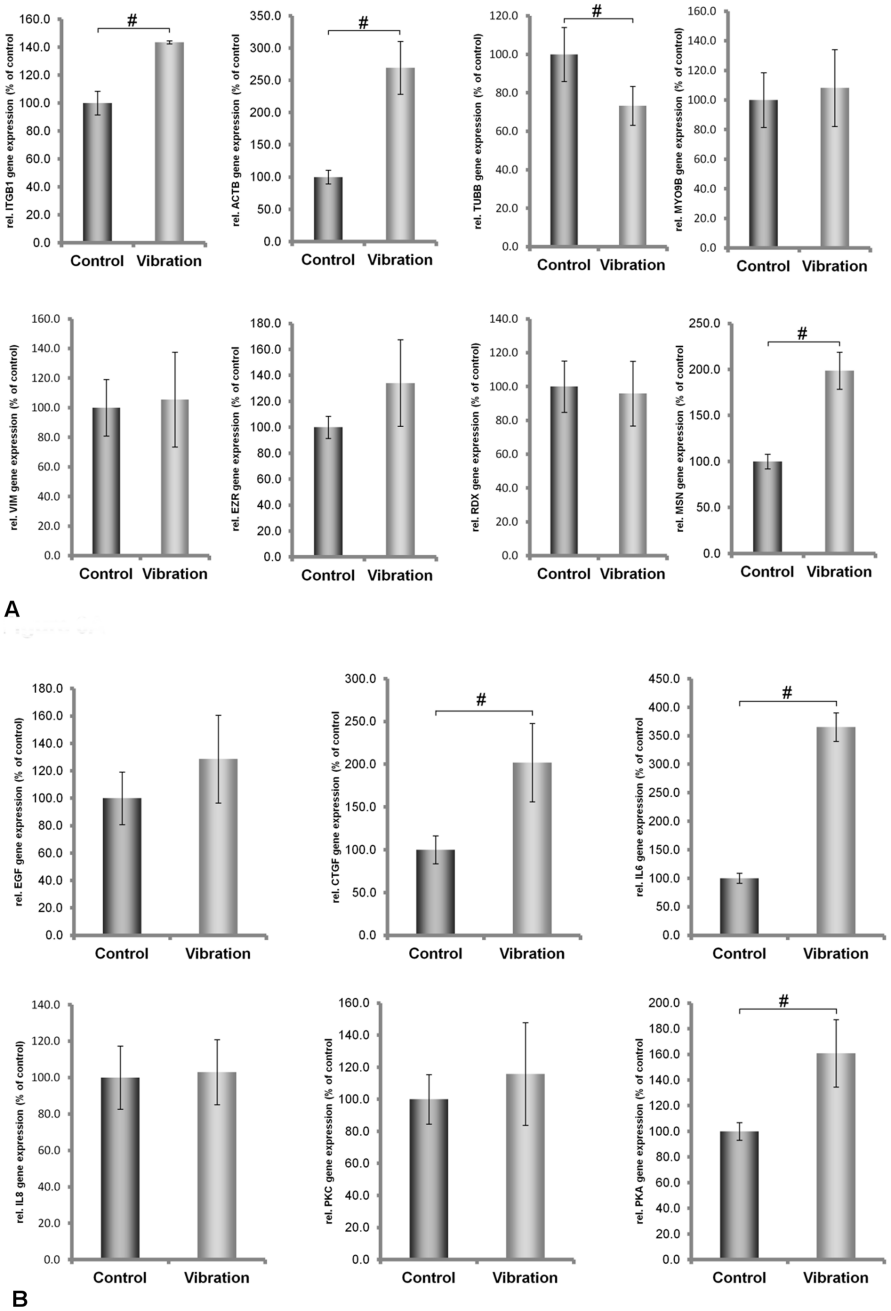
Quantitative real-time PCR showing the alterations in ACTB, MYO9, TUBB, VIM, TLN1, ITGB1, EZR, RDX, MSN, IL-6, IL-8, EGF, CTGF, PRKAA1 and PRKCA gene expression after vibration treatment of CGTH W-1 cells.

Both cell lines continued to grow during the vibration treatment lasting two hours. Afterwards, microscopic observation revealed that cell attachment still persisted. However, determination of mRNA concentrations of the proteins demonstrated that ML-1 and CGTH-W1 cells were affected differently by vibration. While only *IL6* mRNA concentrations decreased in ML-1 cells, the concentrations of the other investigated transcripts remained unchanged (Fig. 2). In CGTH W-1 cells, the mRNAs of *CTGF*, *IL6*, *ACTB*, *MSN, ITGB1* and *PRKAA1* were upregulated, while *TUBB* mRNA was down-regulated. All the other mRNA levels were not affected by vibration (Fig. 3). Hence, ML-1 cells appeared to be more resistant against vibration than CGTH-W1 cells.

### Differential Gene Expression Induced by Short-term Hypergravity

**Figure 4:**
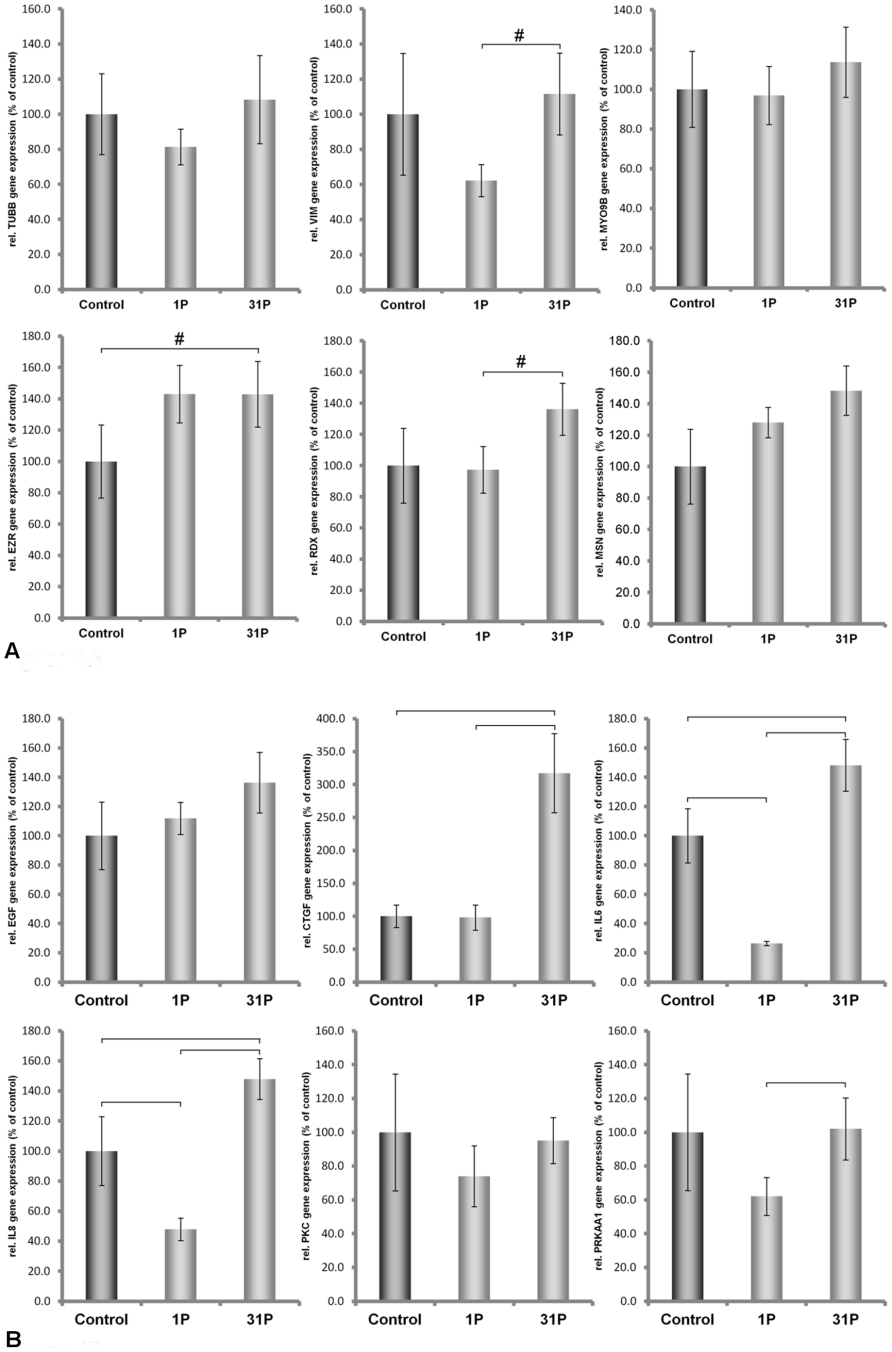
Quantitative real-time PCR showing the alterations in MYO9, TUBB, VIM, EZR, RDX, MSN, IL-6, IL-8, EGF, CTGF, PRKAA1 and PRKCA gene expression after P1 and P31 hypergravity treatment of ML-1 cells.

During a parabolic flight, cells are exposed to vibrations as well as to hypergravity. In earlier flight experiments [14], [15], we observed that major cellular changes occurred during the first parabola. Therefore, here, we exposed the cells to an acceleration profile that occurs during the first parabola. Even during these short periods (2 x 20 seconds) of centrifugation, significant alterations in the transcription of our genes of interest occurred. In ML-1 cells, we detected significant down-regulation of mRNA concentrations of *IL6* and *IL8*, whereas no significant change was observed for *TUBB, MYO9, VIM, EZR; RDX; MSN, EGF, CTGF, PRKCA,* and *PRKAA1* (Fig. 4).

**Figure 5:**
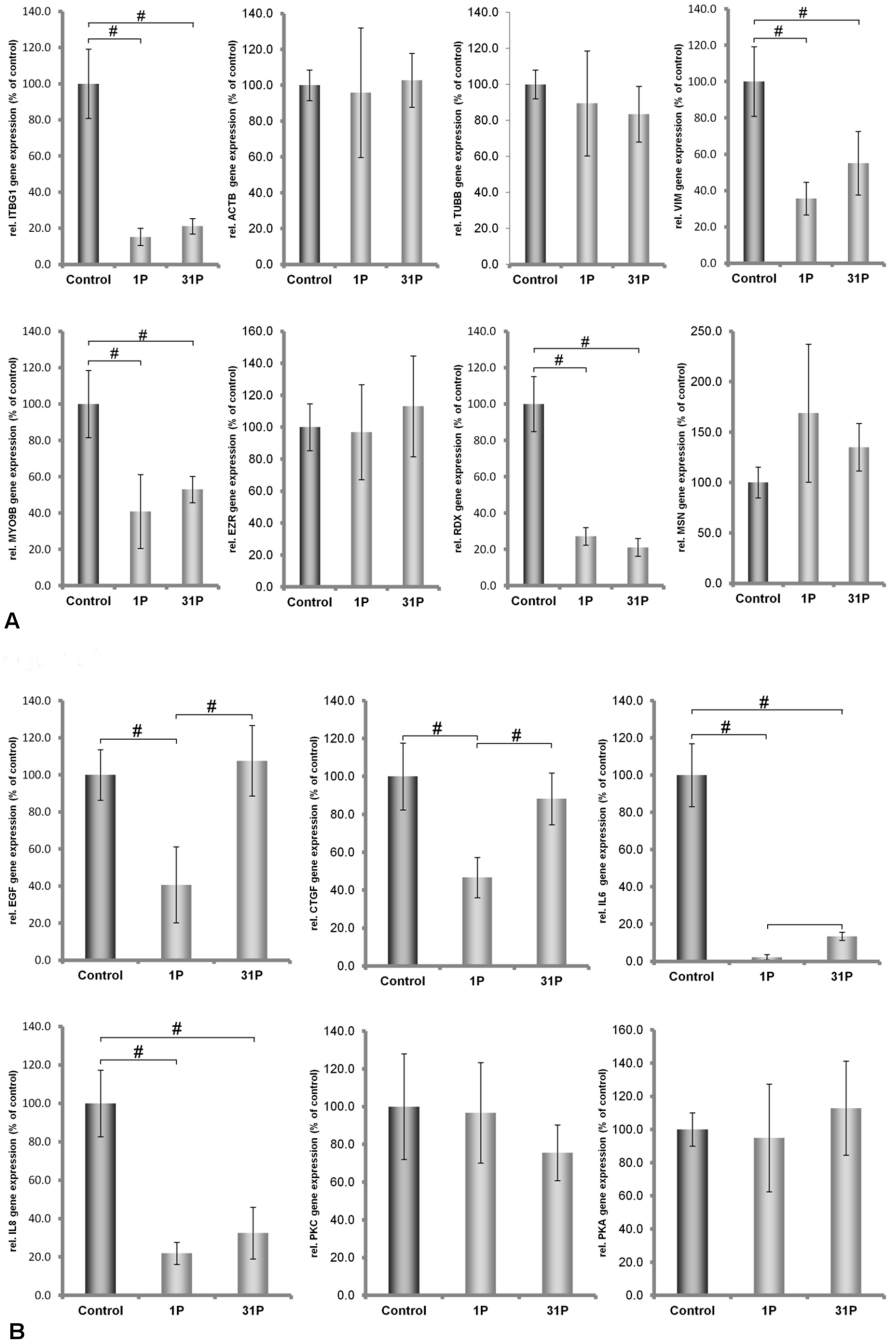
Quantitative real-time PCR showing the alterations in ACTB, MYO9, TUBB, VIM, TLN1, ITGB1, EZR, RDX, MSN, IL-6, IL-8, EGF, CTGF, PRKAA1 and PRKCA gene expression after P1 and P31 hypergravity treatment of CGTH W-1 cells.

In CGTH W-1 cells, the mRNAs of *CTGF, IL6, IL8, ITGB1, VIM, TLN1, MYO9B* and *RDX* were downregulated, while the concentrations of the other mRNAs tested remained un-affected (Fig. 5). Again, ML-1 cells appeared more resistant than CGTH W-1 cells, especially with respect to cytoskeletal proteins.

### Differential mRNA Expression Induced by Repeated Exposure to Hypergravity

Although the major effect of a parabolic flight is seen after the first parabola, we exposed the cells to hypergravity that occurs during a total of 31 parabolas and examined the mRNA levels afterwards. Repeated exposure to hypergravity reversed the effects of the first parabola on *IL6* and *IL8* mRNA levels in ML-1 cells. Now, *IL6* and *IL8* transcripts were up-regulated, together with *CTGF, EZR* and *RDX* mRNAs (Fig. 4).

In the CGTH W-1 cell line, the expression of *ITGB1, VIM, MYO9, RDX, IL6,* and *IL8* decreased after P31, while the concentrations of the other seven types of mRNA remained unaffected (Fig. 5). These results demonstrated that the hypergravity that occurs during 31 parabolas mainly up-regulates the transcription of our target-genes in ML-1 cells, but down-regulates them in CGTH W-1 cells.

### Changes in Intracellular Protein Concentrations Induced by the Vibration or Hypergravity that Occurs during 31 Parabolas

**Figure 6:**
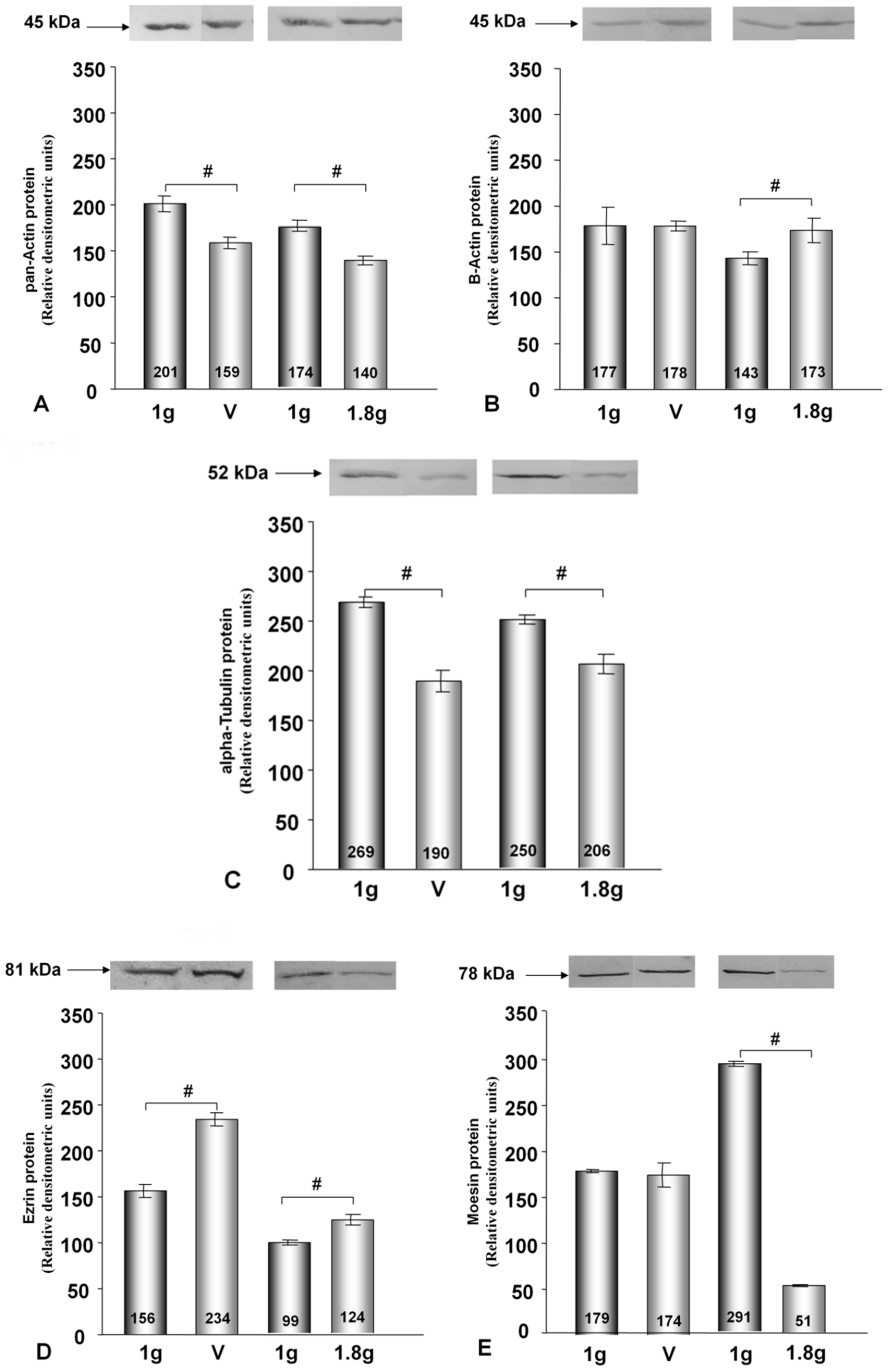
Western Blot analyses showing the changes in pan-actin, alpha tubulin, beta-actin, ezrin and moesin protein levels after vibration and P31 hypergravity treatment of ML-1 cells.

**Figure 7:**
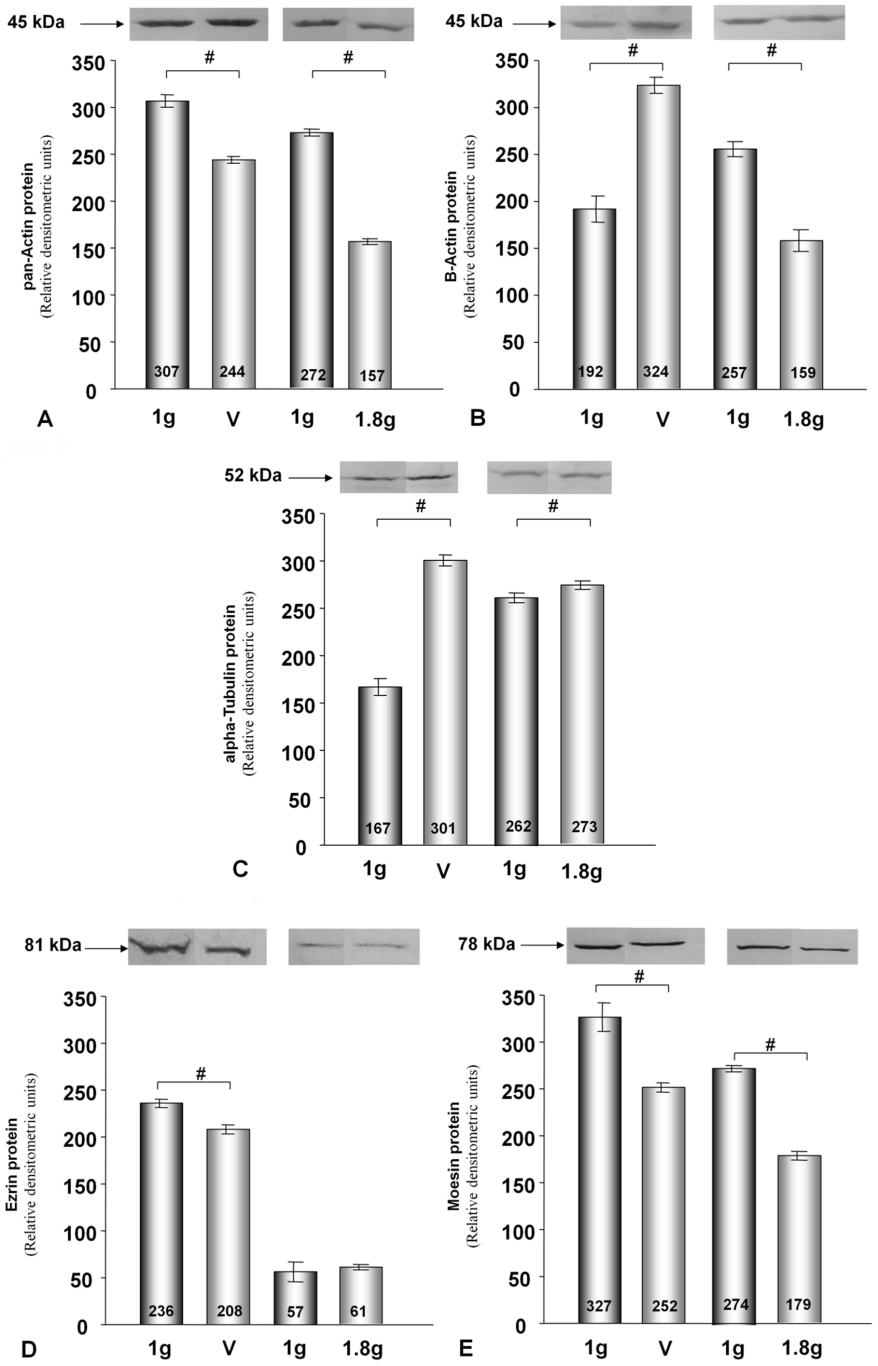
Western Blot analyses showing the changes in pan-actin, alpha tubulin, beta-actin, ezrin and moesin protein levels after vibration and P31 hypergravity treatment of CGTH W-1 cells.

Published data on the correlations between intracellular mRNA levels and protein concentrations are inconsistent [22]–[24]. Therefore, we investigated the protein concentrations of actin, tubulin, moesin and ezrin, in addition to the mRNA concentrations. Using an antibody that binds to all variants of actin chains (pan-actin), we observed that pan-actin protein concentrations were reduced in each cell line after each type of treatment when compared to 1 *g* control cells (Fig. 6A, 7A). Protein levels of alpha-tubulin chains were different in ML-1 and CTGH W-1 cells. In ML-1 cells, the protein concentrations decreased after vibration treatment and hypergravity exposure, while the converse was observed in CGTH W-1 cells (Fig. 6C and 7C). In these cases, a direct comparison between protein and mRNA concentrations was not possible because various genes encode the proteins tagged by the antibodies. When we used an antibody directed against beta-actin chains only, which are encoded by the *ACTB* gene, we detected no change in ML-1 cells after vibration treatment and increased concentrations after exposure to hypergravity. In CTGH W-1 cells, up-regulation of this protein was noted after vibration and a down-regulation following hypergravity exposure (Fig. 6B and 7B). Thus, protein and mRNA changes corresponded well in CGTH W-1 cells after exposure to vibration (Fig. 3, 7B), but not following repeated hypergravity (Fig. 5, 7B). There was also a correlation in ML-1 cells between protein and mRNA changes in response to vibration (Fig. 2), but not to hypergravity exposure (Fig. 4) [14]. In addition, Western blot analyses were performed for moesin and ezrin. In ML-1 cells, only ezrin was influenced by vibration, while moesin and ezrin were affected by hypergravity (Fig. 6D and 6E). In CGTH W-1 cells, both types of proteins were down-regulated by vibration, but only moesin protein expression decreased under hypergravity (Fig. 7D and 7E). In these cases, protein and mRNA concentrations corresponded only in three of the eight analyses (Fig. 3–7).

## Discussion

To study the possible influence of vibration or hypergravity on thyroid cancer cells, we selected proteins that are involved in maintaining or modulating cell structures. The reason for choosing them was due to the earlier observations that these proteins react most sensitively when the cells are exposed to microgravity [8], [10], [12]. In addition, we aimed to find a direct protein/gene target of the mechanical forces that in turn triggered changes in other proteins [25].

The proteins whose genes we investigated have different cellular functions. Actin, myosin, tubulin, and vimentin are the major components of the cytoskeleton, supporting and reinforcing the cell structure [26]. Ezrin and moesin belong to the ERM family, which also includes radixin. These proteins can interact with both plasma membrane proteins and filamentous actin [27], and regulate the organization and dynamics of the actin cytoskeleton in general [28]. Attached to actin filaments via talin, integrin penetrates the cell membrane and interacts with the extracellular matrix surrounding the cells. This link is required to transmit signals from the cell environment to the interior [29]. Epithelial growth factor (EGF), connective tissue growth factor (CTGF) and the interleukins 6 and 8 are produced by thyroid cells and act on their cytoskeleton in an autocrine manner [30].

In the present study, comparison of protein and related mRNA concentrations revealed poor correlation with regard to beta-actin, ezrin and moesin. This may be explained by the recent finding that cellular abundance of proteins is predominantly controlled at the level of translation [31]. Still, gene expression data can provide important information about cell structure variations induced by various stimuli.

Protein kinase C alpha belongs to the protein kinase C family and is a regulator of the cytoskeleton [32], [33]. At the protein level, it is activated by the kinase mTORC2 [34]. PKC-alpha gene expression can be modified by chemicals, growth factors and hormones [35]. In our system, the physical forces applied to the cells did not influence this enzyme. In many systems, PKC-alpha activates the gene expression of IL-6 [36], [37], which is a multifunctional cytokine expressed by human thyrocytes [38], [39]. We observed that *IL6* expression was modified, while PKC-alpha gene expression remained unaffected. Hence, we concluded that the observed changes in *IL6* mRNA levels occurred independently of PKC alpha. It is known from the literature that different regulatory mechanisms are responsible for *IL6* gene expression [40]. In FRTL-5 thyroid cells, *IL6* mRNA expression is enhanced by mechanisms involving the cAMP/PKA pathway [41]. Furthermore, mechanical stress or stretching enhances IL-6 production in human lung epithelial cells and smooth muscle cells via NFκB [42], [43]. To the best of our knowledge, it was unknown until now whether or not biomechanical stress induced IL-6 production in thyroid cells. We did previously observe that *IL6* gene expression was enhanced in FTC-133 thyroid cancer cells, which remained adherent for 24 hours on the RPM [12]. In endothelial cells, IL-6 sensitivity to simulated microgravity was observed. *IL6* gene activation was higher in adherent and tube-forming cells after 5 days on the RPM than in 1 *g* control cells. During the following two days, *IL6* mRNA concentration decreased in adherent cells, but increased in tube-forming cells [44]. These changes correlated well with those in the IL-6 protein levels, as higher quantities of IL-6 proteins were secreted into the culture supernatant when the endothelial cells were incubated on the RPM for 24 hours compared to the control cells. This effect was abolished in the presence of bFGF [45].

From a scientific point of view, however, it is even more interesting that mRNA concentrations of growth factors varied more sensitively under the influence of the mechanical forces generated by hypergravity and vibration than the mRNA levels of proteins that build or modulate the cytoskeleton directly. *IL6* levels changed under each treatment. Besides *EZR* in ML-1 cells and *TUBB* in CGTH-W1 cells, all the transcriptional changes occurred in the same direction as the alteration of the *IL6* gene. Moreover, changes in IL-6 protein amounts in culture supernatants were observed earlier, when endothelial cells were cultured under altered gravity conditions on an RPM [45]. Thus, we conclude that IL-6 plays an important role when mechanical forces act on human thyroid cells *in vitro*. If this conclusion is true, it would explain why so many gene alterations are observed after cells have been exposed to microgravity [46], while whole organisms are influenced moderately during comparable periods of exposure. In humans, *IL6* gene expression is down-regulated by hormones such as estrogen and testosterone [40]. Hormones controlling *IL6* expression are absent when isolated cells are cultured *in vitro*.

In the future, it will be important to take these effects into account when conducting experiments in real microgravity. Especially those setups with longer or more phases of hypergravity and vibration, such as parabolic flights, will be more affected by them. For longer missions in Space on board the ISS hypergravity should not be a factor, but a certain level of vibration originating from the different machines as well as from the astronauts themselves (for example during workout) will also always be present and needs to be considered. Using different cells, our group has recently tried to analyze the impact of hypergravity and vibration on the overall effect of altered gene expression during parabolic flights [47], and those first results seem to suggest, that while hypergravity and vibration induce opposite effects from those of microgravity in the cells, weightlessness is the overall stronger stimulus. However, more experiments have to be conducted to refine these results and they also have to be supplemented by long-term studies. These data would be of high importance for our future Spaceflight experiment (thyroid cancer cells in Space) on the ISS in November this year.

Taken together, we observed an influence of vibration and hypergravity on the gene expression of thyroid cancer cells. Remarkably, the two types of cells reacted differently. ML-1 cells appeared more resistant against vibration than CGTH-W1 cells. Therefore, control experiments on appropriate centrifuges and vibration devices are necessary to interpret data obtained from the microgravity experiments.
